# Immunotherapy and Targeted Therapies in Treatment of Visceral Leishmaniasis: Current Status and Future Prospects

**DOI:** 10.3389/fimmu.2014.00296

**Published:** 2014-06-26

**Authors:** Om Prakash Singh, Shyam Sundar

**Affiliations:** ^1^Infectious Disease Research Laboratory, Department of Medicine, Institute of Medical Sciences, Banaras Hindu University, Varanasi, Uttar Pradesh, India

**Keywords:** immunotherapy, visceral leishmaniasis, treatment, resistance, IL-10, anti-IL-10 mAb

## Abstract

Visceral leishmaniasis (VL) is a vector-borne chronic infectious disease caused by the protozoan parasite *Leishmania donovani or Leishmania infantum*. VL is a serious public health problem, causing high morbidity and mortality in the developing world with an estimated 0.2–0.4 million new cases each year. In the absence of a vaccine, chemotherapy remains the favored option for disease control, but is limited by a narrow therapeutic index, significant toxicities, and frequently acquired resistance. Improved understanding of VL pathogenesis offers the development and deployment of immune based treatment options either alone or in combination with chemotherapy. Modulations of host immune response include the inhibition of molecular pathways that are crucial for parasite growth and maintenance; and stimulation of host effectors immune responses that restore the impaired effector functions. In this review, we highlight the challenges in treatment of VL with a particular emphasis on immunotherapy and targeted therapies to improve clinical outcomes.

## Introduction

Leishmaniasis, a spectrum of diseases caused by *Leishmania* species, affects ~12 million people around the world, mostly in developing countries. It is transmitted by sand flies (*Phlebotomus* species) as extracellular flagellated promastigotes and replicate as intracellular, aflagellated amastigotes in mononuclear phagocytes in mammalian host (1). Depending on the species, the disease symptoms may range from self-healing skin lesions to the fatal visceral form known as kala-azar or visceral leishmaniasis (VL). Kala-azar is the most severe form of the leishmaniasis and accounts for 200–400 thousands new cases and over 50,000 deaths annually (2). Anthroponotic transmission of VL is caused by *Leishmania donovani* and prevails in Indian subcontinent and East Africa; while zoonotic transmission of VL is caused by *L. infantum* (syn. *L. chagasi*) in the Mediterranean region, South America, and Southwest and Central Asia. The majority of all cases (90%) are found in India, Nepal, Bangladesh, Brazil, South Sudan, and Ethiopia (3), where transmission typically occurs from humans infected with kala-azar or post kala-azar dermal leishmaniasis (PKDL) (4, 5). More specifically, an estimated 80% of the global burden of VL occurs in South Asia (e.g. in 2007, 100,000–150,000 of the cases occurred in India alone). The situation is especially severe in Bihar State in eastern India, where some districts have faced the worst epidemic since the 1970s. Left untreated, VL is fatal, and the burden of disease expressed in disability-adjusted life years is estimated to be ~2.5 million. Furthermore, over 90% of all individuals with VL earn an income of <2 United States (US) dollars per day. Because VL is associated with resource-poor regions, access to care is another challenge in the overall management and treatment of VL (6, 7). The situation is further complicated by the emergence of resistant strains to currently available anti-leishmanial drugs and by the limited availability of inexpensive, non-toxic drugs (Table 1). Antimonial chemotherapy has been the mainstay for VL treatment for more than 50 years, and continues to be the recommended first line treatment in most parts of the world (8). Resistance to pentavalent antimonials (Sb^v^) has first been reported in northern Bihar, where nearly 60% of individuals are now unresponsive to this drug (9). Pentamidine has been the second line drug used in Sb^v^ refractory patients. Unfortunately, its efficacy has also declined over the years, and now curing only ~70% patients. Resistance has also been reported with pentamidine and miltefosine (10, 11), and there is growing concern for resistance with paromomycin monotherapy (12). Increasing parasite drug resistance, longer treatment times, and associated toxicity to patients has resulted in the need to use more expensive drugs such as AmBisome^®^ (liposomal amphotericin B) and miltefosine (8). A recent study demonstrated a single dose of liposomal amphotericin B is an effective VL treatment (13). However, concerns about emerging drug resistance with single drug therapy have led to testing liposomal amphotericin B in combination with oral miltefosine (14, 15). This strategy is still requiring administration of the drugs over an extended period and cost and toxic side effects are major issues. Hence, dose-sparing strategies that shorten treatment times are likely to be of major benefit to VL treatment programs. In addition, an intervention that can reduce the risk of developing PKDL is also highly desired. Importantly and relevant to this discussion, drug therapy works most effectively with help from the host immune system, and in particular, cell mediated immune (CMI) responses. Hence, immune modulation that stimulates immunity and work synergistically with drugs has enormous potential for drug-sparing strategies that would help in the treatment of a broad range of diseases.

**Table 1:** **Current VL treatments with anti-leishmanial drugs: their mode(s) of action on parasites, dosage, efficacy, advantages and limitations**.

	Drugs	Mode(s) of action	Dosage	Efficacy (%)	Advantages	Limitations	Reference
1	Pentavalent antimonials: sodium stibogluconate (Pentostam) or meglumine antimoniate (Glucantime)	Acts as pro-drug that is converted to active and more toxic trivalent form within the amastigote/macrophage; and this active trivalent SbIII form inhibits trypanothione reductase and exposes parasite to oxidative stress of the host	20 mg/kg/day (i.m.) for 20–30 days in India	80–90 (50% in Bihar, India)	Low cost and easily availability in endemic area	Pancreatitis, cardiac arrhythmias, acquired resistance in the Indian subcontinent	(8, 16, 17)
2	Amphotericin B (Fungizone)	Form complexes and bind to ergosterol in parasite membranes that create pores, which alter ion balance, increase membrane permeability resulting in cell death; also acts as an inhibitor of ergosterol biosynthesis	0.75–1.0 mg/kg for 15–20 infusions either daily on alternate days in India (i.v)	>95%	Effective in antimony resistant regions, primary resistance is unknown	High cost and need of prolonged hospitalization, rigor, and fever with renal complications, hypokalemia	(16, 18, 19)
3	Liposomal amphotericin B (AmBisome)	Targeted delivery of drug to the infected macrophage and mechanism of action is same as amphotericin	3.0 mg/kg/day for 5 days (total 15 mg) OR 10 mg/kg as a single dose, i.v	>96%	Highly effective, low toxicity, resistance is not documented	High cost	(13)
4	Paromomycin (aminoglycoside antibiotic), also known as aminosidine	Exact mechanism is not known. In bacteria, inhibits protein synthesis, but in *Leishmania*, it decreases the mitochondrial membrane potential of *L. donovani* promastigotes	11 mg/kg of base/day for 21 days (i.m.)	95%	Acts synergistically with antimonials, effective, well tolerated, and cheapest drug for VL	Reversible ototoxicity but no nephrotoxicity, lack of efficacy in East Africa	(20 –22)
5	Miltefosine	Interacts with the cell membrane of *Leishmania* parasites by modulation of cell surface receptors, inositol metabolism, and phospholipase activation, Cell death being mediated by apoptosis	50 mg/day for adults <25 kg and 100 mg/day >50 kg adults (oral)	85–95%	First oral drug for VL. Currently first line of treatment in Indian subcontinent	Potentially teratogenic, vomiting, and diarrhea with occasional hepatic and renal toxicity	(15, 19)
6	Pentamidine	Accumulate in parasite mitochondria and inhibit mitochondrial topoisomerase II, binding to AT-rich sites in the minor groove of DNA followed by inhibition of transcription process	4 mg/kg/day for three times weekly for 15–20 dose (i.m or i.v)	70–80%	Low efficacy, toxic. May be used in combination with other drugs	Gastrointestinal side effects, cardiac, arrhythmias, hypotension, pancreatitis, and irreversible insulin-dependent diabetes mellitus	(23, 24)

Currently, there is no effective human vaccine available for any form of leishmaniasis. One of the major challenges in vaccine development has been a limited understanding of the precise immune mechanisms required for controlling parasite growth (25, 26). In the present review, we highlight the current status and challenges in treatment of leishmaniasis with focus on immune based strategy for improving treatment regimens for VL.

## Immune Regulation and Immunopathogenesis

Mammals have evolved to recognize and control pathogens, including the recognition of infected cells. This is achieved by the coordinated actions of innate and adaptive immune mechanisms [reviewed in Ref. (27)]. The innate immune response involves the recognition and early control of threats to the body as well as for the activation of adaptive immunity. Adaptive immune response involves B cells that produce specific antibodies; and T cells that recognize peptide antigens. T cell responses are mediated by CD8^+^ T cells that recognize peptides derived from both inside and outside of cells and presented by major histocompatibility class (MHC) I molecules on the cell surface or CD4^+^ T cells that recognize peptides from microbes or antigens engulfed by professional phagocytes and then presented on the context of MHC II molecules. The main targets of immunomodulatory strategies should be CD4^+^ T cells because they play critical roles in coordinating immune responses by producing molecules critical for the production of high affinity antibodies by B cells, essential for activation of CD8^+^ T cells to kill infected and transformed cells.

Based on the studies in the *L. major*/BALB/c mouse model, the immune dysregulation associated with non-healing and disseminating forms of leishmaniasis has been associated with a parasite-driven Th2 polarized response, in which interleukin (IL)-4 is especially dominant [reviewed in Ref. (28)]. Accumulating data in human VL, however, indicate that the cytokine responses are not highly polarized, and even during the acute phase of disease, elevated levels of interferon-γ (IFN-γ) mRNA have been found in lesional tissue, such as the spleen and bone marrow (29–31). Furthermore, in human VL, overproduction of IL-10 provides a much better correlate of susceptibility than IL-4. The vast array of cytokines, chemokines, and immune mechanisms involved in the host immune response to *Leishmania* clearly highlights the complexity of diseases (32, 33). Based on studies in mice, production of interleukin-12 (IL-12) by antigen-presenting cells (APCs) and IFN-γ by T cells appear to be required for the control of the parasites and development of acquired resistance (34, 35). IL-12 is regulatory cytokine for initiation and maintenance of the Th1 response and plays an important role in the induction of IFN-γ production by T and NK cells (36–40). Priming of susceptible BALB/c mice with exogenous rIL-12 during *Leishmania* infection also promotes protection and gives self-healing phenotype (41, 42). On the other hand, *Leishmania* parasites have been shown to inhibit IL-12 production, resulting in decreased leishmanicidal activity of macrophage (43). Maintenance of the proportion of CD4^+^ and CD8^+^ T cells required for cytokines secretion is the crucial step in generation of immunity against leishmaniasis. In active VL, both CD4 and CD8 cells are activated and play distinct but cooperative role in disease resolution. CD4^+^ cells play a role in the control of primary infection, while CD8^+^ cells are thought to be more important during secondary immune response (44).

Human VL is characterized by very high titers of *Leishmania*-specific antibodies, appearing soon after infection but before the development of cellular immunological abnormalities (45, 46). These anti-leishmanial antibodies persist up to 16 years after treatment, suggesting its possible involvement in immunity (47). There are reports that B cells and antibodies correlate with pathology, but role of these antibodies in disease resolution or protection is unknown. Studies have also shown that animals lacking B cells are resistant to *Leishmania* infections (48), but such evidence on human VL are still lacking. Importantly, in endemic area of Bihar (India), strong association were found between seropositivity and progression to clinical diseases in healthy individual (49), suggesting its role in disease pathogenesis. Therefore, in-depth studies are required before any conclusion can be drawn. More recently, we have reported high anti-leishmanial antibodies in Indian VL patients compared to Sudanese patients and could be one of the factor for lower sensitivity of serological tests in East Africa (50). Most importantly, anti-leishmanial antibodies do not play any role in antigen-specific IFN-γ or IL-10 production in whole blood of active VL patients (unpublished data).

## Targeted Therapy and Immunotherapy

In the absence of human vaccine and effective vector control measures, chemotherapy is the only option for treatment and control of VL. Several hundred comparative and prospective cohort studies on therapies for leishmaniasis have been published (Table 1). Increasing evidence of drug unresponsiveness and resistance has raised concern to save the drugs, as the armory of anti-leishmanial drugs is limited. Reports of emerging resistance to Miltefosine, the newest and only oral anti-leishmanial drugs, which is the basis of VL elimination program, are particularly worrying (14); and makes VL management and elimination challenging. Drug discovery is struggling to prevent resistance, therefore changes in the drug policy are much needed step as on today. Reductions in VL morbidity and mortality will require the development and deployment of immune modulators in order to achieve the prophylactic or therapeutic goal; and also prevent the transmission of *Leishmania* from human to sand fly. One of the most interesting approaches currently being explored is immunotherapy and targeted therapy [reviewed in Ref. (51)]. Targeted therapies act by blocking essential biochemical or signaling pathways that are indispensable for *Leishmania* parasite growth and survival, however, immunotherapy involves the use of biological molecules or compounds to modulate immune responses in combination with drugs. Over the last two decades, various approaches of immunotherapies or targeted therapies have been developed and applied in the treatment of human leishmaniasis (Table 2). The strengths and weaknesses of such therapies suggest that both approaches might have complimentary roles in VL treatment, and combination could prove synergistic. Because targeted therapies can induce rapid parasite clearance, with a consequent decrease in *Leishmania* associated immune-suppression, they might afford a favorable window for immunotherapy to improve the efficacy of treatment.

**Table 2:** **Immunotherapy of human leishmaniasis**.

Country	Year	Immunotherapeutic agent	Chemotherapeutic	No. of	Disease/	Treatment	Reference
			agent	patients	parasite	efficacy	
India	1995	IFN-γ	Sb^v^	16	VL	87%	(52)
Brazil	1990	IFN-γ	Sb^v^	17	VL	82.3%	(53)
Brazil	2005	GM-CSF	Sb^v^	05	CL	100% Cure	(54)
Brazil	2006	Killed *L. amazonensis* + BCG	Glucantime	47	ACL	87%	(55)
Brazil	2006	Mixed antigens^a^	–	06	MCL	76–94%	(56)
Brazil	2002	Killed *L. amazonensis*	Meglumine	47	ACL	100%	(57)
Argentina	2011	Killed *L. amazonensis* + BCG	–	01	ACL	High	(58)
Peru	2007	Imiquimod	Sb^v^	07	CL	72%	(59)
Kenya	1993	IFN-γ	Sb^v^	10	VL	75%	(60)
Sudan	2008	Alum/ALM + BCG	Sb^v^	15	PKDL	87%	(61)
Iran	2006	Imiquimod	Glucantime	59	CL	44.1%	(62)
Uzbekistan	1993	Leukinferon (i.m.)	Monomycin	50	CL	High	(63)
Venezuela	1990–1999	Pasteurized *L. braziliensis* + BCG	–	5341	CL	91.2–98.7%	(64)
Venezuela	1994–2000	Mixture antigens^b^	Sb^v^	87	CL	Moderate	(65)
Venezuela	2004	Pasteurized *L. braziliensis* + BCG	–	07	MCL, DCL	100%	(66)

VL, visceral leishmaniasis; CL, cutaneous leishmaniasis; MCL, mucocutaneous leishmaniasis; DCL, diffuse cutaneous leishmaniasis; PKDL, post kala-azar dermal leishmaniasis; BCG, bacillus Calmette–Guerin; Sb, sodium stiboguconate; IFN-γ, interferon-γ; mixture antigens

^a^: TSA, thiol-specific antioxidant; LmSTI1, *L. major* stress inducible protein 1; LeIF, *Leishmania* elongation initiation factor; Lbhsp83, *Leishmania* heat shock protein 83; GM-CSF, granulocyte macrophage colony-stimulating factor; mixture antigens

*^b^: amastigotes from *L. (L.)amazonensis* (La), *L. (L.)venezuelensis* (Lv), *L. (V.)brasiliensis* (Lb), and *L. (L.)chagasi* (Lch) Tosyl-Lysyl Chloromethyl-ketone (TLCK) treated and Non-idet P-40(NP-40) extracted (VT)*.

## Targeting Host Immunity by Anti-Leishmanial Drugs/Molecules

Within the mammalian host, *parasites* reside as amastigotes in phagocytic cells such as polymorphonuclear neutrophils (PMN), macrophages, and dendritic cells (DCs). Therefore, it is important to identify an immunomodulatory compound with leishmanicidal properties capable of activating phagocytic cells. Following entry of *Leishmania* parasite into the mammalian host, PMNs are thought to be the first effector cells recruited to the site of infection within 24 h, implying that they possibly serve as host cells for *Leishmania* parasites in the very early phase of infection (67). Neutrophils being inherently short-lived and apoptotic, are usually cleared without triggering activation of macrophages (67), while *Leishmania* parasites are known to delay neutrophils apoptosis, possibly by interfering with production of reactive oxygen species (ROS) (68, 69). Therefore, it would be logical and important to search an anti-leishmanial compound capable of generating an oxidative burst within *Leishmania* infected neutrophils to effectively eliminate parasites. Berberine chloride has been one of the compounds recently reported to enhance the apoptosis of *L. donovani*-infected neutrophils via modulation of the MAP kinase pathways (70).

*Leishmania* parasites that enter into macrophages via the uptake of infected, apoptotic PMNs may survive and multiply effectively (67). Since, macrophages have ability to kill parasite upon activation, *Leishmania* parasites overcome these macrophage activation and recognition by creating an anti-inflammatory milieu, beneficial for parasites survival. It has been reported that the amount of TGF-β secreted by macrophages following uptake of infected PMNs is higher than after direct uptake of *L. major* promastigotes (67), suggests that uptake of infected, apoptotic PMNs are responsible for creation of this environment within macrophages. Therefore, targeting pathogens residing in neutrophils should be taken into consideration when designing targeted novel anti-leishmanial compounds, as neutrophils harbor and transport parasites. For example, antimonials (sodium stibogluconate) increase the phagocytic capacity of neutrophils along with increased production of superoxide (71), unfortunately the loss of efficacy of antimonials has occurred in the Indian subcontinent and thus raised concern to search another compounds. In fact, several strategies to interfere with macrophage signaling by parasites have been reported that favor its survival in host cells (72). Oghumu et al. have highlighted the role of STAT4 pathway in immunity to *L. donovani* infection and also reported the evidence that STAT4 is dispensable for antimonial-based chemotherapy (73). Furthermore, some of the other strategies employed by *Leishmania* to evade effector mechanisms of the host immune system are the recruitment of inhibitory CD4^+^CD25^+^FoxP3^+^ regulatory T cells (Treg) (74, 75), inhibition of macrophage phagosomal maturation (76), and inhibition of DC maturation (77). Receptors expressed on Treg or its corresponding ligands on effectors cells, such as glucocorticoid-induced TNF receptor family-related protein (GITR), PD-1, it is ligands programed cell death ligand-1 (PD-L1, B7-H1) or cytotoxic T lymphocyte antigen-4 (CTLA-4) could be also used as potential targets in future studies, as targeting these regulatory pathways has proven effective in experimental VL (78, 79).

## Cytokine Immunotherapy

Cytokines are the messengers of the immune system. They have autocrine and paracrine functions, so that they function locally or at a distance to suppress or enhance immunity. Attempts to identify cytokines that selectively induce Th1 responses might be useful in VL therapy. The evidence of the utility of cytokines as therapeutic use came from the studies by Murray et al., when an anti-IL-10 receptor monoclonal antibody (anti-IL-10R mAb) was reported to inflict parasite killing through an inducible nitric oxide synthase-dependent mechanism (80). Thus, immunostimulatory cytokines (e.g., IFN-γ, IL-12, GM-CSF) or antibodies that target suppressive/deactivating cytokines are being investigated or proposed as monotherapies or as combination therapies with Sb^v^ or other drugs. Combination therapy with recombinant human IFN-γ and pentavalent antimonials have been reported as stronger parasitological and clinical cure; compared with the drug alone in VL patients from Brazil, Kenya, and India (53, 60, 81). Short course of IFN-γ is thought to be sufficient to activate macrophage and thereby accelerate parasitologic effect of Sb^v^. IL-12 is another key cytokine inhibited by *Leishmania* parasites. Exogenous treatment with rIL-12 during *Leishmania* infection leads to resistance in susceptible mice (41), suggesting its important use in clinical outcome. However, suppression of other cytokines, including receptor fusion antagonists of IL-13, IL-4, and TGF-β inhibit parasite replication but only marginally affect parasite clearance without the induction of a synergistic effect with pentavalent antimonials (82). In a study, GM-CSF plus either with meglumine antimonite (54) or a mixture of *L. major* antigens (LmSTI1 + LeIf + HSP83) (56), was reported as being highly effective in treating American CL and MCL (Table 2).

## IL-10: Role in VL Pathogenesis and Immunotherapy

Visceral leishmaniasis pathogenesis has been associated to an overproduction of the regulatory cytokine, IL-10, which can promote parasite replication and disease progression. Several studies performed to characterize the immunologic effects of VL have focused on the role of IL-10 in the suppression of DC functions and rendering macrophages unresponsive to activation signals (83). Experimental models have demonstrated that IL-10 plays a central role in the pathogenesis and parasite growth in VL, as IL-10-deficient BALB/c and C57BL6 mice are highly resistant to *L. donovani* infection (84). Treatment of *L. donovani*-infected wild-type mice with a single dose anti-IL-10R mAb and daily low doses of Sb^v^ resulted in rapid control of the *L. donovani* infection and dramatically enhanced the therapeutic effects of Sb^v^ namely, an over 10-fold dose-sparing effect was observed with Sb^v^ and a shortened duration of treatment (85). In a separate study, single dose anti-IL-10R mAb (0.5 mg) treatment triggered a 63% liver parasite killing in *L. donovani*-infected BALB/c mice; moreover, when administered at a reduced dose (0.1 mg), the anti-IL-10 mAb enhanced the effect of Sb^v^, also administered at a suboptimal dose (50 mg/kg), leading to a 72% liver parasite killing (82). Similar results were observed in *L. donovani*-infected BALB/c mice treated with a suboptimal single dose (0.1 mg) of an anti-IL-10R mAb and low-dose Amphotericin B (2 mg/kg total dose) (86). The combination therapy induced a 76% liver parasite killing, compared with a 16% observed with the anti-IL-10R mAb alone.

Elevated levels of IL-10 in serum as well as enhanced IL-10 mRNA expression in lesional tissue during active disease are a consistent finding in human VL [reviewed in Ref. (87)]. More recently, we have reported antigen stimulated IL-10 production in whole blood cells of VL patients and have shown a strong association of IL-27 and IL-21 with the up-regulation of the IL-10 response, and revealed the presence of both IFN-γ and IL-10 producing antigen-specific cells in the peripheral blood of VL patients (74, 88, 89). The findings have led to an underlying hypothesis that during active disease antigen-specific IL-10 producing T cells are activated under conditions that also drive strong and persistent Th1 responses, and the balance of these cells and the cytokines they produce favors the progression of disease. It has been shown that infected macrophages, Th1, Th2, CD8^+^ T cells, and subsets Treg, of which naturally occurring CD4^+^CD25^+^Foxp3^+^ Treg cells and antigen-inducible or adaptive Treg are the best defined, are all a potential source of IL-10 capable of suppressing *Leishmania*-specific immunity (90–92). Key findings have identified CD4^+^CD25^−^Foxp3^−^ or adaptive Treg as the main source of both elevated IL-10 and IFN-γ in the spleen of VL patients (74). Furthermore, antigen driven IL-10 production has been difficult to detect in culture of peripheral blood mononuclear cells (PBMCs) (88, 93, 94). These findings are consistent with reports from a number of studies, which suggest that the immunologic defect in VL is characterized not by the complete absence of a potentially curative type 1 immune response, but by the co-expression of suppressive cytokines that compromise the leishmanicidal function and potency of the effector response in target organs, such as the spleen. A direct role for IL-10 in the pathology of VL is supported by studies demonstrating that IL-10 blockade can enhance IFN-γ responses (29, 95). More recently, we have demonstrated anti-parasitic effect of IL-10 blockade in human VL, showing that neutralization of IL-10 results in marked reduction of parasite number present in splenic aspirate cells (89). In continuation with these *ex vivo* supporting findings, Phase I study of anti-IL-10 mAb alone and in combination with AmBisome have been recently proposed for the human trial (clinicaltrial.gov) and this combination is expected to induce synergistic effects that contain the VL infection and immunopathology associated with the disease, while overcoming the threat of drug resistance and possibly achieving a chemotherapeutic dose-sparing effect that results in better efficacy and adherence to treatment. Importantly, demonstrating a therapeutic benefit from the IL-10 neutralization as a proof of concept will open the door to other strategies targeting the inhibition of IL-10 and other immunosuppressive factors.

## Dendritic Cell-Based Immunotherapy

Another novel approach is the application of DCs for the induction of antigen-specific T cell immunity. The interaction of DCs and *Leishmania* parasites are complex and thought to be responsible for control of infection or progression of clinical disease (96). DCs play an important role in initial anti-*Leishmania* T cell responses and promoting their differentiation into memory T cell to achieve long lasting immunity, which makes them attractive candidates for potential synergy with immunotherapy [reviewed in Ref. (51)]. Interestingly, a C-type lectin receptor, DC-SIGN (DC-specific ICAM-3-grabbing non-integrin), which is exclusively expressed on tissue monocyte-derived DCs, has been shown to favor parasite survival by binding with distinct *Leishmania* species. It is then suggested that this receptor could also be taken into consideration as therapeutic target for both visceral and cutaneous leishmaniasis (97).

Dendritic cells based immunotherapy combined with antimony-based chemotherapy has been shown very effective against murine VL (98). Bone marrow derived DCs pulsed with soluble *L. donovani* antigen when given in combination with antimonials has been shown to reduce both hepatic and splenic parasite burden significantly (51). Thus, the future of DC-based immunotherapy appears promising and it could be looked upon as a prospective vaccine against VL.

## Challenges and Future Directions

Treatments that enhance immune responses to fight against diseases are of significant clinical interest. A possible approach to overcome some of the challenges associated with the management and treatment of VL is the use of immune based combination therapy (99), which has been proven successful in other parasitic diseases, such as malaria, tuberculosis, and leprosy (100). A combination of drugs with different modes of action could eliminate the potential for drug resistance and induce a chemotherapeutic dose-sparing effect, since the mechanism for resistance would be different for each drug (100). One drug could target the parasite itself, while a second drug or compound could modulate the immune system of the host (101–103). Likewise, the combination of drugs with different half-lives could provide a synergistic effect in the timing and exposure of the parasite to the different drug levels (100, 103).

Although, considerable progress on VL treatment has been made over the past years, we still have a limited understanding of the precise immune mechanism underlying human VL. One of the major problems in translating discoveries from disease models into treatments for humans is the risk that potential treatment strategies do not work on human cells in the same way as they do in the experimental model. Second and most important key issue for immunotherapy or targeted therapies is whether intensified anti-*Leishmanial* effects can be achieved without a corresponding increase in serious toxicities, as immunomodulatory agents that provoke an immune response may also pose a risk of severe sensitization, which might be anticipated to increase allergic reactions and lead to reduction in treatment efficacy.

Cytokine (e.g., IL-10) has therapeutically been used as a recombinant protein (i.e., a large molecule), which is quite expensive to produce. It can be only administered by injection, which is also quite inconvenient for the patient. It will then be important to ensure that the cost associated with cytokine immunotherapy must be less than conventional treatment and reach to the populations that need it most. Another better approaches could be to target the molecules acting downstream of the cytokine receptors or signal transduction. The problems in such cases are the specificity, as the known cytokine signaling pathways are shared by different cytokines. Therefore, problems and side effects associated with the use of cytokine therapy have to be addressed properly before its clinical application.

Although, these observations strongly support immunotherapy as a promising alternative to conventional chemotherapy against VL, big challenge remains to ensure long term maintenance of response and safety of treatments with biologic agents.

## Conclusion

Each VL patients represents our failure to prevent leishmaniasis, and each death represents our failure to treat soon enough. Until VL elimination has been achieved, drug treatment will remain crucial to prevent complications and death from VL. There is an urgent need for innovative and effective alternative therapies against VL. Understanding of crucial cellular pathways that promote *Leishmania* parasite growth and maintenance together with the development of compounds or agents that specifically inhibit these pathways has offered a new era for anti-leishmanial therapy. The use of immunotherapy and targeted therapy could aid in addressing some of the current challenges associated with the management and treatment of VL, namely, minimizing resistance to currently available drugs, improving the therapeutic index, decreasing the dose or length of treatment, and reducing the cost of therapy. With the emergence of targeted delivery systems and technology to block the IL-10 transcription and other relevant molecules involved in the IL-10 signaling (e.g., STAT3), a new era of molecular targeting of regulatory cytokines is on the horizon.

## Conflict of Interest Statement

The authors declare that the research was conducted in the absence of any commercial or financial relationships that could be construed as a potential conflict of interest.
